# How can we monitor the impact of national health information systems? Results from a scoping review

**DOI:** 10.1093/eurpub/ckz164

**Published:** 2019-10-24

**Authors:** Marie Delnord, F Tille, L A Abboud, D Ivankovic, H Van Oyen

**Affiliations:** 1 Department of Epidemiology and public health, Sciensano, Brussels, Belgium; 2 Department of Medical Sociology and Rehabilitation Science, Charité Berlin University of Medicine, Berlin, Germany; 3 Department of Public Health, Amsterdam University Medical Centers, Amsterdam, The Netherlands; 4 Division of Health Informatics and Biostatistics, Croatian Institute of Public Health, Zagreb, Croatia; 5 Department of Public Health and Primary Care, Ghent University, Ghent, Belgium

## Abstract

**Background:**

National health information (HI) systems provide data on population health, the determinants of health and health system performance within countries. The evaluation of these systems has traditionally focused on statistical practices and procedures, and not on data use or reuse for policy and practice. This limits the capacity to assess the impact of HI systems on healthcare provision, management and policy-making. On the other hand, the field of Knowledge Translation (KT) has developed frameworks to guide evidence into practice.

**Methods:**

A scoping review of the KT literature to identify the essential mechanisms and determinants of KT that could help monitor the impact of HI systems.

**Results:**

We examined 79 publications and we identified over 100 different KT frameworks but none of these were focused on HI systems per se. There were specific recommendations on disseminating evidence to stakeholders at the institutional and organizational level, and on sustaining the use of evidence in practice and the broader community setting.

**Conclusions:**

We developed a new model, the HI-Impact framework, in which four domains are essential for mapping the impact of national HI systems: (i) HI Evidence Quality, (ii) HI System Responsiveness, (iii) Stakeholder Engagement and (iv) Knowledge Integration. A comprehensive impact assessment of HI systems requires addressing the use of HI in public health decision-making, health service delivery and in other sectors which might have not been considered previously. Monitoring Stakeholder Engagement and Knowledge Integration certifies that the use of HI in all policies is an explicit point of assessment.

## Introduction

National health information (HI) systems compile evidence on population health, the determinants of health and health system performance within countries (i.e. HI).^1–3^ HI is usually collected in routine from population health registers, health facilities, and also during national health surveys. Benchmarks of population characteristics, health and service needs are useful for research, decision-making and intervention in public health policy and practice.^3^^,^^4^ For example, we know that in Europe, there is up to 4-fold variation in stillbirth and infant mortality,^5^^,^^6^ persistent disparities in cancer mortality,^7^ and gender-based patterns in survival and disability^8^; knowledge of these inequalities has been used to inform more global frameworks that aim to ensure health for all (i.e. Sustainable Development Goal 3). In 2016, the World Health Organization (WHO) presented an action plan to strengthen the use of evidence for policy-making in the European Region.^9^^,^^10^ The current Joint Action on Health Information, InfAct, which gathers 40 public health institutes from 28 European countries also embodies this will for a more integrated HI strategy.^3^^,^^4^ Whereas the vision is clear, it requires monitoring the impact of national HI systems on reducing the evidence-to-practice gap.^11^ In this context, we define impact as the demonstrated use of evidence by stakeholders for decision-making and intervention in public health policy and practice.^1^^,^^12^^–14^

The Performance of Routine Information System Management (PRISM) framework was seminal in identifying the key features of efficient HI systems, including the use of information for decision-making.^15^ Yet, evaluations of these systems have traditionally focused more on statistical data processes and data quality, and less on how data are integrated to practice.^1^ On the other hand, the field of Knowledge Translation (KT) has developed frameworks that guide ‘the appropriate exchange, synthesis and ethically sound application of knowledge to interventions that strengthen the healthcare system and improve health’.^16^^,^^17^ Therefore, our aim with this study was to draw from the lessons learned in the field of KT in order to derive a new HI system evaluation framework—one based on monitoring the impact of national HI systems in healthcare, policy-making and service delivery, as well as in other sectors which might not have been considered previously.

## Methods

### Strategy

This article builds on the work done on the evaluation of HI systems by the WHO and the United States Agency for International Development (USAID).^18–20^ HI systems contribute to sound decision-making at all levels of the health system.^2^ End-users have different roles ranging from providing care, implementing screening programmes, planning interventions and policy-making. Therefore, the scope of our study bridges across different fields of practice which warranted a system-wide approach, and a broad search strategy. We conducted a scoping review of KT frameworks; this methodology answers far-reaching questions, and is more amenable than systematic reviews to explore the conceptual boundaries of a given topic.^21^ We investigated PubMed, Medline and the Cochrane Database of Systematic Reviews between August and December 2018, using common terms used to define KT in the literature. Straus et al.^22^ note that ‘the terms implementation science or research utilization are common in Europe, while in the United States, the terms dissemination and diffusion, research use, knowledge transfer, and uptake are often used. In Canada, the terms knowledge transfer and exchange and knowledge translation are employed’. We built our search using the following terms combined and truncated*: knowledge*, translation, health data*, dissemination, implementation, adaptation, evidence informed*, evidence based*, public health, health policy, healthcare, impact, health system*, performance, health information*, tool, framework, monitoring, evaluation (Supplementary annexe S1). We further examined the grey literature and websites of public health knowledge brokers (i.e. the Canadian National Collaborating Centre for Methods and Tools, the US Agency for Healthcare research and Quality, and Knowledge Translation Australia). Reference tracking was applied, and our bibliography was managed in RefManager 12.

### Eligibility criteria

We focused on reviews of KT frameworks and interventions published between 2008 and December 2018. We used Nilsen’s taxonomy of KT frameworks for screening publications^23^ according to process-based (i.e. on the mechanisms and stages of evidence-use), or determinant-based frameworks (i.e. on the barriers and facilitators to evidence-use) operating at organizational and/or institutional level. Per standard scoping review methodology, we included publications even when the risk of bias was not explicitly assessed.^21^ We excluded, however, study protocols, commentaries, editorials and opinion pieces, or when the full-text article could not be retrieved (Flow chart in Supplementary annexe S1).

### Data charting and analysis

Publications were essentially screened by the first author based on the title, the abstract and the full text. Inclusions and charting were independently compared for accuracy and completeness by the co-authors (D.I., F.T., H.V.O., and L.A.A.). From each source, we extracted the following:

- Characteristics: author and citation; country of origin; publication year; and the number of studies/frameworks included in the review.
- Content: Type of framework (e.g. KT processes/determinants), socio-ecological level (e.g. organization, community, systems); KT stage (e.g. dissemination and/or implementation), and field-use (e.g. community health, health policy and healthcare).

We conducted a thematic content analysis which consisted in examining (i) the mechanisms, and (ii) the determinants underpinning the use of evidence in practice. We defined practice as the act of intervening (directly or conceptually) in healthcare provision, service planning and delivery, policy-making and evaluation.

## Results

In total, we screened 533 publications, and we included 79 publications on the mechanisms and determinants of KT. Reviews came primarily from the USA, Canada, Australia (65%) and the UK (22%). Across publications there were large differences in the terminology that was used—with respect to how evidence is defined (e.g. data, knowledge, research, innovations and ideas), or embedded into practice (e.g. knowledge uptake, transfer, exchange, circulation and brokering). Although our search spanned the last 10 years, most reviews were published in the last 5 (67%). There were many more KT publications in healthcare (53 studies), versus policy (16) or community health (8), and two reviews in public health in general. We saw an evolution in the publications, with a greater emphasis on KT in healthcare in the beginning of the study period, and over time an increasing focus on the use of evidence in health policy, and ultimately at the level of health system. This could indicate a growing trend in recognizing the importance of interdisciplinary interventions in KT, as also noted by Vollmar et al.^24^

The studies we included examined between 4^25^ and 159^26^ different KT frameworks, and as shown in table 1, there were 45 reviews on the process of KT.^17^^,^^22^^,^^23^^,^^25–65^ In general, KT can be summarized as a dynamic and iterative cycle in which data-providers ‘*push*’^9^ information into the hands of a target audience—which is known as the dissemination stage.^28^ In turn, data-users can choose to ‘*pull*’^9^ this evidence for intervention in a particular setting (i.e. health facility, local community)—which is known as the implementation stage.^28^ Feedback from the field guides further practice changes, and informs future data needs thereby completing the process. In our study, there were 19 reviews on the dissemination of evidence to stakeholders, 26 on the implementation of evidence into practice. Another 34 reviews were focused on both aspects of dissemination and implementation highlighting their interdependency in the KT process. Some KT frameworks were applied in specific areas of health, namely: cancer research,^48^^,^^49^^,^^58^^,^^66^^,^^67^ child health and welfare,^38^^,^^57^^,^^63^^,^^68^^,^^69^ chronic illness,^26^^,^^53^^,^^70^^,^^71^ cardiovascular health,^72^ healthy aging,^41^^,^^54^^,^^73^ immunization,^74^ acute and primary care,^27^^,^^71^^,^^75^ mental health,^37^^,^^38^^,^^51^^,^^68^^,^^73^ nursing,^76^ physiotherapy,^61^ chiropractic,^77^ rehabilitation,^39^ renal replacement therapy^53^ and genomics.^47^

**Table 1:** Reviews of knowledge translation process frameworks and interventions, in public health policy and practice, from 2008 to 2018

Authors, ref	Title	Country	Year	Type of framework	KT stage^a^	Field	Socio-ecological level^b^	No. of studies	No. of frameworks
Armstrong et al.^25^	Knowledge translation strategies to improve the use of evidence in public health decision making in local government: intervention design and implementation plan	Australia	2013	Process	I	Public health; policy	Systems	4	
Ben et al.^27^	Effective strategies for scaling up evidence-based practices in primary care: a systematic review	Canada, Australia	2017	Process	I	Healthcare primary care	Organizations	14	
Best et al.^65^	Building knowledge integration systems for evidence-informed decisions.	Canada	2009	Process	D&I	Healthcare; policy	Organizations		5
Brown et al.^28^	An Overview of Research and Evaluation Designs for Dissemination and Implementation.	USA	2017	Process	D&I	Research; policy; healthcare	Systems		8
Brownson et al.^29^	Fostering more-effective public health by identifying administrative evidence-based practices: a review of the literature.	USA	2012	Process	I	Policy; administrative	Systems	144	
Burchett et al.^30^	How do we know when research from one setting can be useful in another? A review of external validity, applicability and transferability frameworks.	UK	2011	Process	I	Public health	Systems	38	35
Contandriopoulos et al.^31^	Knowledge exchange processes in organisations and policy arenas: a narrative systematic review of the literature	Canada	2010	Process	D&I	Organizations and policy-makers	Organizations, systems		205
Darzi et al.^32^	A methodological survey identified eight proposed frameworks for the adaptation of health related guidelines.	Lebanon, USA, Canada	2017	Process	D	Healthcare	Organizations		8
Damschroder et al.^56^	Fostering implementation of health services research findings into practice: a consolidated framework for advancing implementation science.	USA	2009	Process/Determinants	I	Healthcare	Systems		19
Davison et al.^34^	Critical examination of knowledge to action models and implications for promoting health equity	Canada	2015	Process	D&I	Healthcare; policy	Organizations, systems		48
Davies et al.^33^	Mobilising knowledge to improve UK health care: learning from other countries and other sectors – a multimethod mapping study.	UK	2015	Process/Determinants	D&I	Healthcare; social care and education	Systems	71	
Escoffery et al.^35^	A systematic review of adaptations of evidence-based public health interventions globally	USA	2018	Process/Determinants	I	Community health	Systems		42
Gagnon et al.^57^	A Systematic Review of Knowledge Translation (KT) in Pediatric Pain: Focus on Health Care Providers.	Canada	2016	Process/Determinants	D&I	Healthcare	Organizations	98	
Gardois et al.^36^	Health promotion interventions for increasing stroke awareness in ethnic minorities: a systematic review of the literature.	UK	2014	Process	I	Healthcare	Systems	18	
Goldner et al.^37^	A narrative review of recent developments in knowledge translation and implications for mental health care providers	Canada	2014	Process/determinants	D&I	Healthcare: child mental health	Organizations	61	
Hack et al.^58^	Facilitating the implementation of empirically valid interventions in psychosocial oncology and supportive care	Canada	2011	Process/Determinants	I	Healthcare: cancer	Systems	3	28
Hanson et al.^38^	The what, when, and why of implementation frameworks for evidence-based practices in child welfare and child mental health service systems	USA	2016	Process/Determinants	I	Healthcare: child welfare and mental health	Systems		9
Jones et al.^39^	Translating knowledge in rehabilitation: systematic review	USA	2015	Process/Determinants	I	Healthcare: rehabilitation	Organizations	26	
Kneale et al.^59^	The use of evidence in English local public health decision-making: a systematic scoping review.	UK	2017	Process/Determinants	D&I	Public health; policy	Systems	23 papers from 21 studies	
Leeman et al.^40^	What strategies are used to build practitioners' capacity to implement community-based interventions and are they effective?: a systematic review	UK	2015	Process	I	Healthcare, community health	Systems	42 papers from 29 studies	
Leeman et al.^131^	Developing Theory to Guide Building Practitioners' Capacity to Implement Evidence-Based Interventions.	USA	2017	Process	I	Public health	Systems		24
Lourida et al.^41^	Dissemination and implementation research in dementia care: a systematic scoping review and evidence map.	UK	2017	Process/Determinants	D&I	Healthcare: geriatrics	Organizations	88	
Mairs et al.^42^	Online strategies to facilitate health-related knowledge transfer: a systematic search and review.	Canada	2013	Process/Determinants	D	Community health	Systems	45	
Matus et al.^43^	Research capacity building frameworks for allied health professionals - a systematic review.	Australia	2018	Process	D	Healthcare: allied health professionals	Organizations	6	
Meyers et al.^44^	The quality implementation framework: a synthesis of critical steps in the implementation process.	USA	2012	Process	I	Healthcare; community health	Systems, organizations		25
Milat et al.^45^	Narrative review of frameworks for translating research evidence into policy and practice	Australia	2017	Process	D&I	Healthcare and policy	Systems		41
Moullin et al.^46^	A systematic review of implementation frameworks of innovations in healthcare and resulting generic implementation framework.	Australia	2015	Process	I	Research, policy, healthcare	Systems		49
Nilsen et al.^23^	Making sense of implementation theories, models and frameworks	Sweden	2015	Process	I	Public health	Systems	38	
Payne et al.^47^	Translational informatics: enabling high-throughput research paradigms.	USA	2009	Process	D&I	Healthcare: genomics	Organizations, systems		
Rajan et al.^48^	Critical appraisal of translational research models for suitability in performance assessment of cancer centers.	The Netherlands	2012	Process	D&I	Healthcare: cancer	Organizations		12
Senore et al.^49^	How to enhance physician and public acceptance and utilisation of colon cancer screening recommendations	Italy	2010	Process/Determinants	D&I	Healthcare: cancer, community health	Organizations, systems	NA	
Slade et al.^50^	Frameworks for embedding a research culture in allied health practice: a rapid review	Australia	2018	Process	D&I	Healthcare	Organizations		16
Stander et al.^61^	Training programmes to improve evidence uptake and utilisation by physiotherapists: a systematic scoping review.	South Africa, Australia	2018	Process	D&I	Healthcare: physiotherary	Organizations		10
Stirman et al.^51^	Bridging the Gap Between Research and Practice in Mental Health Service Settings: An Overview of Developments in Implementation Theory and Research.	USA	2016	Process	I	Healthcare: mental health setting	Organizations		73
Straus et al.^22^	Knowledge translation is the use of knowledge in health care decision making	Canada	2011	Process/determinants	D&I	Healthcare	Organizations		NA
Strifler et al.^26^	Scoping review identifies significant number of knowledge translation theories, models, and frameworks with limited use.	Canada	2018	Process	D&I	Healthcare: cancer and chronic diseases	Organizations	596	159
Tabak et al.^52^	Bridging research and practice: models for dissemination and implementation research.	USA	2012	Process	D&I	Research	Systems		61
Van der Veer et al.^53^	Translating knowledge on best practice into improving quality of RRT care: a systematic review of implementation strategies.	Netherlands	2011	Process	I	Healthcare: renal placement therapy; chronic care	Organizations	93	
Van Eerd et al.^54^	Knowledge brokering for healthy aging: a scoping review of potential approaches.	Canada	2016	Process	D&I	Healthcare: healthy aging	Organizations	19	
Ward et al.^62^	Developing a framework for transferring knowledge into action: a thematic analysis of the literature.	UK	2009	Process	D&I	Public health	Organizations, systems		28
Whitney et al.^27^	Evaluation of health information outreach: theory, practice, and future direction.	USA	2014	Process/determinants	D	Public health	Systems	33	
Welch et al.^55^	Health equity: evidence synthesis and knowledge translation methods	Canada	2013	Process	D	Research	Systems	34	
Wilson et al.^64^	Disseminating research findings: what should researchers do? A systematic scoping review of conceptual frameworks.	UK	2010	Process	D	Research	Systems		33
Wilson et al.^63^	Knowledge translation studies in paediatric emergency medicine: A systematic review of the literature.	Canada	2016	Process	D	Healthcare: child health	Organizations	15	
Yost et al.^17^	Knowledge translation strategies for enhancing nurses' evidence-informed decision making: a scoping review.	Canada	2014	Process	I	Healthcare: nursing	Organizations	274	

**Notes**: In Table 1, we are only listing publications focused on KT processes (45 out of 79); publications focused on KT determinants are included in tables 2 and 3.

a D: Dissemination (targeted outreach to stakeholders with evidence): I: Implementation (use of evidence for intervention in a specific setting).

b Socio-ecological level: “systems”, if more than one organizational level is involved: that is health policy and health care.

A common starting point in the KT frameworks we reviewed was the generation of high-quality data.^9^^,^^22^^,^^28–31^^,^^33^^,^^34^^,^^36^^,^^37^^,^^45^^,^^52^^,^^54^^–56^^,^^64^^,^^78–80^ We identified three main attributes of data quality: statistical accuracy, content relevance and coverage.^9^^,^^11^ Standardized methodological approaches, data innovations and advanced statistical methods can improve the accuracy of evidence provided to stakeholders.^67^ On the other hand, content relevance is ensured by providing the data needed by stakeholders for planning, care or service delivery (i.e. information on the burden of disease, or on cost effectiveness and feasibility).^33^^,^^81^ Adequate coverage in the data provides for more equity in the interventions.^9^^,^^30^^,^^36^^,^^82^ For instance, disaggregated data by age, gender or socio-economic status are needed to plan actions that target vulnerable segments of the population, diverse communities or subgroups at the highest health risk.^67^^,^^83^^,^^84^

We found that although data production initiates the KT process, dissemination carries it through. Jones et al.^39^ note that strategies range from passive methods such as printed educational material to more active multicomponent strategies involving audit and feedback from local opinion leaders, while only relying on printed material is regarded as a ‘reasonably ineffective KT strategy’. Two methods, however, have been recognized by Blessing et al.^9^^,^^39^ as particularly useful: (i) Exchange methods in which data-providers and policy-makers for instance work in partnership, as well as (ii) Integrated methods ‘where a knowledge translation platform is institutionalized in an organization or in the broader health system’. When stakeholders have access to evidence, this can spearhead problem recognition and the development of meaningful solutions in the field.^9^^,^^24^ These include actions whether legal, financial or political that align with national and international health targets.^25^^,^^38^^,^^59^^,^^81^^,^^85–^^88^

During the implementation stage, Davies et al.^33^ emphasize that there are three broad types of knowledge use: instrumental (i.e. for direct application in the field), conceptual (i.e. for problem-solving and agenda setting) and symbolic (i.e. in support of predetermined positions or after the decision has already been made). Donaldson et al. suggest monitoring intermediate shifts in stakeholders’ knowledge, attitude and skills^89^ as proof that evidence has been accessed and used,^90^ granted few frameworks provide specific KT outcome or impact measures.^91^^,^^92^

Although in general, KT frameworks share the aforementioned characteristics, we found notable differences in the intricacy of the data-provider/data-user interaction across publications.^45^^,^^52^ For instance in the prominent Re-aim framework, there are five dimensions to consider: reach (R) and effectiveness (E), operate essentially at individual-level; whereas adoption (A), implementation (I) and maintenance (M) are focused on organizational and community-level change.^45^ Another highly cited model, the Knowledge-to-Action framework is based on two components: (i) the Knowledge creation funnel in which research data is generated, synthesized and contextualized to become more useful, and (ii) the Action cycle which includes a range of actions targeting changes in stakeholders’ level of understanding, behaviours and attitudes.^45^ Other models explicitly rely on the latest developments in health information technology for the dissemination and implementation stages. For instance, in the Learning Healthcare Systems framework, electronic health records and data mining facilitate exchanges between data-users and data-providers; this model also puts emphasis on shared decision-making between patients and care providers with the aim to bring quality improvements in clinical care.^93^

There were 58 reviews which explored the determinants of the use of evidence in healthcare, public health policy-making and service delivery. Among which, six were focused on the tools (i.e. checklists and guidelines) which have been developed to facilitate the access to, and the use of evidence.^9^^,^^16^^,^^17^^,^^80^^,^^94^^,^^95^ We have summarized KT determinants by field of practice (e.g. healthcare, policy-making and community health) in tables 2 and 3. Studies insisted on lifting technical (i.e. availability and access to evidence), behavioural (i.e. motivation and competence), organizational (i.e. culture of evidence-use) and contextual KT barriers (i.e. political and social)^11^^,^^15^^,^^33^^,^^35^^,^^41^^,^^54^^,^^59^^,^^66^^,^^81^^,^^87^^,^^88^^,^^96–98^ as shown in table 2. Whereas in table 3, we identified attributes that promote KT and the successful dissemination of evidence such as: (i) clarity of the data/evidence, (ii) timeliness, (iii) a tailored reporting format based on stakeholders’ preferences and (iv) the use of tools to facilitate access to research (i.e. packaging, application, dissemination and communication tools).^9^^,^^41^^,^^42^^,^^54^^,^^56^^,^^59^^,^^64^^,^^67^^,^^80^^,^^81^^,^^96^^,^^99–^^101^

**Table 2:** Technical, behavioural, organizational and contextual barriers to KT in public health policy and practice

Decision-makers	Technical	Behavioural	Organizational	Contextual
Community health managers	Lack of access and availability of applicable research evidence^59^ Lack of timely research output^81^^,^^87^ Lack of credibility of the evidence^81^^,^^97^	Negative perceptions of research utilization: i.e. perceived as a bureaucratic/timely process^59^^,^^96^ Lack of joint understanding between researchers and decision-makers^88^^,^^96^^,^^98^ Lack of motivation, awareness, and skills to seek, appraise, and interpret the evidence/systematic reviews^96^ Formal evaluation of KT activities seen as highly challenging^33^	The culture of decision-making (i.e. resistance to change)^11^^,^^87^ Practical constraints^87^ Lack of accountability in the use of evidence^87^	Competing influences on decision- making^87^, role of the media^98^ ^a^Lack of cultural appropriateness, accept ability or applicability in practice^35,41,66,96^ ^a^Conflicts of interest^87^ ^a^Few agencies involving users/general public in KT activities^33^
Health policy-makers	Lack of access and availability of applicable research evidence^87^^,^^96^ Lack of timely research output^81^^,^^87^ Lack of credibility of the evidence^81^^,^^97^	Negative perceptions of research utilization: i.e. perceived as a bureaucratic/timely process^96^^,^^96^ Lack of motivation awareness, and skills to seek, appraise, and interpret systematic reviews^96^ (i.e. belief that it is too time consuming)^96^^,^^11^ Formal evaluation of KT activities seen as highly challenging^33^	The culture of decision-making (i.e. resistance to change)^11^^,^^87^ Lack of resources or organizational support^11^^,^^81^^,^^100^ Lack of accountability in the use of evidence^87^	^a^Lack of relevance to policy needs (i.e. locally useful, evidence on costs)^98^^,^^81^ ^a^Legislative constraints^81^^,^^97^ ^a^Short-sighted considerations of political support or feasibility^97^ Competing influences on decision-making^87^^,^^104^^,^^105^ Role of the media^98^
Clinicians and allied health professionals	Lack of database access^109^ Lack of rapidly available, and suitably filtered information^22^ ^a^Complex nature of some evidence-based therapies or guidelines^72^	Lack of skills to appraise, understand and apply research evidence (including digital skills)^22^ Lack of awareness or agreement with the guidelines^22^; ^a^Familiarity or confidence in the effectiveness of a particular evidence-based therapy^72^ ^a^Limited skills or competence to use a particular therapy^72^; Knowledge practice gaps^77^	Time, ^a^clinical workload and other pressures^41^. Insufficient capacity for implementation^38^ Organizational resistance to change^70^^,^^72^^,^^73^; ^a^inadequate peer support, organizational or institutional level support^72^^,^^22^^,^^41^ Lack of infrastructure, limited medical facilities to support evidence uptake^72^	^a^Patient needs, preferences and adherence to treatment when these interfere with evidence-based recommendations^22^^,^^56^^,^^72^

Notes: KT determinants are categorized by themes (technical, behavioural, organizational and contextual).

a Field specific determinant.

**Table 3:** Technical, behavioural, organizational and contextual solutions for promoting KT in public health policy and practice

Decision-makers	Technical	Behavioural	Organizational	Contextual
Community health managers	Securing access to high-quality evidence: National portal for access to evidence^101^^,^^96^ Clarity, timeliness, and strength of the evidence^96^ Reports distributed through professional organizations or through a clearinghouse^96^ Online strategies: use of wikis, discussion forums, blogs, and social media, virtual communities of practice and conferencing technology^41^^,^^42^, Knowledge exchange portals^101^; electronic communication channels: newsletters containing summaries of current research developed and directly emailed to managers^96^ User-friendly Reporting format: One-page summaries with key messages tailored to the target audience^64^^,^^67^^,^^96^ Clear relevance of the results and factors important for contextualizing the evidence (i.e. potential short and long term outcomes, benefits, harms or risks)^64^^,^^67^^,^^96^ For systematic reviews^96^ Title framed as a question the report Use of white space, no dense text, Limit tables to one page. The methods should be concise Easy to interpret summary of the results and of the risk of bias of individual studies (graphical display) Recipe type guidance Replacing unfamiliar terms or adding definitions to the review	Increased Motivation, skills and competence: Capacity-building, training and continued education^96^: Multicomponent active delivery of information (as opposed to only access to online registry^96^^,^^39^^,^^67^ Grounding KT activities in existing theories of behavioural change^113^ Targeted messaging, educational visits and summaries^132^, Concept mapping^133^ Greater understanding of transferability and applicability of evidence-based recommendations from one setting to another^30^	Continued interactions with data- providers at organizational, institutional level: Partnerships with researchers to facilitate the use of evidence^96^ Cross-sector and interagency learning^97^; collaborative decision-making with other community organizations^69^ Reflection on the conceptual basis of knowledge mobilization activities^33^ Integration of librarians and health information specialists in the organization^96^ Availability of (human) resources, time^69^ Identifying measurable objectives and ensuring that evaluation measures reflect those objectives^113^, ‘Participatory’ evaluations with all relevant stakeholders.^113^ Networking for information sharing^25^	Integration of evidence, expertise, and values and circumstances Theory-guided effort to identify and address the contextual factors most relevant to any particular intervention^31^^,^^40^^,^^41^^,^^88^^,^^97^ Addressing complex environmental factors and including measures of community sustainability and institutional change in the outcomes^113^ ^a^Focus on a new target population, a new setting^35^^,^^41^ ^a^Increased coverage and equity of access to evidence-based interventions^49^ ^a^Cultural appropriateness,^35^^,^^41^ Applicability of the evidence-based intervention at local level^41^^,^^41^^,^^66^ ^a^Outreach to key players in the communities where the interventions would take place, including in non- clinical settings^40^^,^^69^^,^^88^^,^^113^ ^a^Collaboration between community partners,^69^ Networking for information sharing^25^ ^a^Conducting a community assessment prior to finalizing program's specific objectives^113^ ^a^Harnessing the role of media, and social marketing campaigns^66^
Health policy-makers	Securing Access to high-quality evidence: Clarity, timeliness and strength of the evidence^81^^,^^100^^,^^112^ Fast and easy referencing,^101^ Reports distributed through professional organizations or through a clearinghouse,^96^ use of electronic communication channels: i.e. newsletters containing summaries of current evidence^9^ User-friendly Reporting format: One-page summaries with key messages tailored to the target audience^64^^,^^67^^,^^96^ Clear relevance of the results and factors important for contextualizing the evidence (i.e. potential short and long term outcomes, benefits, harms or risks),^64^^,^^67^^,^^96^ For systematic reviews^96^: (1) Title framed as a question the report (2) Use of white space, no dense text, (3) Limit tables to one page. (4) The methods should be concise (5) Easy to interpret summary of the results and of the risk of bias of individual studies (graphical display) (6) Recipe type guidance (7) Replacing unfamiliar terms or adding definitions to the review ^a^Framing the evidence in terms of ‘how’/ solution-oriented recommendations^64^^,^^67^^,^^96^ Providing tools to aid evidence-use^16^^,^^17^^,^^80^^,^^94^^,^^95^: Packaging tools include synthesis methods, such as policy briefs, and visualization methods; Application tools include surveillance data and modelling/simulation to explore the behaviour and performance of processes and interventions; Dissemination and communication tools include health information-sharing platforms, newsletters and person-to-person communications; Linkage and exchange tools such as knowledge networks facilitate the dissemination of health information and the likelihood of translating research to policy^9^^,^^17^^,^^100^	Increased Motivation, skills and competence: Capacity-building, training and continued education^81^^,^^100^ Multicomponent active delivery of information (as opposed to only access to online registry)^39^^,^^67^^,^^96^ Targeting multiple organizational capabilities, including staff skills and competence in using evidence^108^ Grounding KT activities in existing theories of behavioural change, and based on an understanding of how policy agencies use evidence and how they view their roles^108^ A common understanding between data-providers and data-users,^81^ a sense of trust, and a shared vision among stakeholders^75^ Greater understanding of transferability and applicability of evidence-based recommendations from one setting to another^30^ Tailored interactive workshops and goal-focused mentoring^100^	Continued interactions with data-providers at organizational, institutional level: ^a^Stable, clear and decentralized decision-making authority has greater capacity to adopt innovations^102^ Support for the use and evaluation of research use in policy development^25^^,^^96^ allocation of resources^96^ Reflection on the conceptual basis of approaches and increased evaluation of knowledge mobilization activities^33^ Partnerships between researchers and policy-makers/ managers to facilitate the conduct and use of evidence^9^^,^^81^^,^^96^; Co-creation of knowledge with researchers^81^^,^^96^ ^a^ Advisory role by policy-makers on research teams (i.e. involved with the development of research questions, assisted with dissemination)^9^ ^a^Leaders commissioning evidence^9^^,^^100^^,^^103^; research targeted at the needs of decision-makers^100^ Networking for information sharing^25^ Institutionalized knowledge brokers^33^ Integration of librarians and health information specialists in the organization^96^	Integration of evidence, expertise, and values and circumstances Recognition of multi-level processes (professional, organizational, local system) and interactions across these level^75^ The primacy of local evidence, and the important role of local experts^41^^,^^88^ ^a^Harnessing political processes at all levels to shape the selection and use of evidence in decision-making^41^^,^^88^ Networking for information sharing^25^ Taking account of the determinants related to the social-political context in which the evidence is used^31^^,^^97^ ^a^Support to enable cross-sector and interagency learning^33^; Platforms for cross-sector collaborations^112^
Clinicians and allied health professionals	Securing Access to high-quality evidence: Evidence strength and quality^56^ ^a^Rapid access to suitably filtered evidence^109^ Annual reports from quality registers should be more detailed and give more consideration to random variation^41^^,^^99^ Providing tools to aid evidence-use^16^^,^^17^^,^^80^^,^^94^^,^^95^ Packaging tools include synthesis and visualization methods^94^ Application tools include surveillance data and modelling/simulation to explore the behaviour and performance of processes and interventions^16^^,^^9^^,^^41^^,^^94^ ^a^Clinical decision support systems^41^^,^^80^^,^^109^ Dissemination and communication tools include health information-sharing platforms, newsletters and person-to-person communications^16^^,^^41^^,^^94^	Increased motivation, skills and competence: ^a^Health professionals' perceived usability and practice behaviour change vary by type of information and communication technology^41^^,^^75^ Increasing personal readiness for change^70^ Building and maintaining trust^88^ Research capacity building and training programmes^43^^,^^61^; Training in the use of specific technologies supporting access to (i.e. electronic health records), and use of evidence^41^^,^^41^^,^^68^^,^^80^^,^^94^^,^^109^ Bringing out the added-value of websites and search engines^41^^,^^80^ ^a^Trained staff in the teaching and practice of EBM^72^ (Evidence-Based Medicine); understanding of effective KT strategies^37^ Participatory action frameworks based on interactive knowledge exchange (e.g. blended learning) rather than passive unidirectional approaches alone (e.g. lectures)^41^^,^^73^ Involving opinion leaders (person typically nominated by colleagues as ‘educationally influential’)^85^	^a^Improving organizational climate and culture^41^^,^^56^^,^^68^: Agreement of objectives and goals, collaborative decision-making, greater levels of social cohesion^41^^,^^88^ Facilitation to prepare clinicians and organizations for implementation^70^^,^^76^ Multimodal delivery of KT interventions at the organizational level^39^^,^^67^^,^^79^^,^^109^ ^a^Building communities of practice and advanced care planning: i.e. Engaging frontline staff and health managers in data collection and evidence, use^41^^,^^80^, ^a^Availability of peers staff and technical resources,^106^ social networks^110^ Involvement of librarians and health information specialists^96^ Leadership and managerial support in the use of evidence^41^^,^^56^: Fidelity monitoring and supervision of KT efforts^41^^,^^68^ Requiring particular forms of evidence to inform decision-making^41^^,^^75^ Financial incentives and resources^38^ Measuring/improving organizational readiness for change^70^^,^^95^^,^^102^ ^a^Performance rankings^14^ Active follow-up of KT interventions^95^	Integration of evidence, expertise, and values and circumstances^58^: Facilitating the implementation of empirically valid interventions in health care^58^ i.e. Policy measures aimed at supporting screening delivery, as well as organizational changes, influencing the operational features of preventive services^41^^,^^49^ Resources allocated to ensure that participation in evidence-based programmes is not limited by financial barriers^106^ Recognition of multi-level processes (professional, organizational, local system) and interactions across these level^41^^,^^75^ ^a^Pan-regional organizations shape innovation decision-making at lower levels^58^ (i.e. Reference Networks, Professional societies and practice guideline development)

Notes: KT determinants are categorized by themes (technical, behavioural, organizational and contextual) and subthemes which are underlined.

a Field specific determinant.

In table 3, we have listed further recommendations for engaging stakeholders at the organizational and institutional level in using evidence, and adopting best practices.^56^ In policy, a setting that has a ‘stable, clear and decentralized decision-making authority’ is said to have a greater capacity to adopt innovations.^102^ When leaders commission or champion evidence themselves this can facilitate the KT process.^9^^,^^100^ Inter-sectorial structures (i.e. the EU Joint Research Centre) and institutionalized knowledge brokers can also play a role in disseminating evidence in a whole-of-government approach.^33^ A common understanding between data-providers and data-users, a sense of trust, a shared vision among stakeholders and the provision of resources also facilitate KT (table 3). Nonetheless, we also found that there are many competing sources of information which can interfere with KT. At the level of the decision-maker, testimonials and narratives can sometimes be considered more convincing for policy-making,^103^ as well as advice from personal contacts, sometimes mid-level managers with no direct expertise in public health.^104^^,^^105^

In medical facilities, Reschovsky et al.^106^ state that the extent to which KT occurs can vary by clinical indication, specialty and health service setting (i.e. operational size, structure: private vs. public). We found that engaging frontline staff and health managers in improving data collection and its use is crucial for team transformation and quality improvement^107^; capacity building and workforce development also.^31^^,^^43^^,^^61^^,^^70^^,^^72^^,^^81^^,^^95^^,^^108^ A culture of evidence-use can also be supported by health information technology to facilitate a rapid access to evidence (i.e. e-journals, clinical decision support systems).^41^^,^^68^^,^^80^^,^^94^^,^^109^ Local opinion leaders,^85^ greater social cohesion in medical facilities,^41^^,^^80^^,^^106^^,^^110^ performance rankings,^14^ providing resources and financial incentives further contribute to KT at organizational level.^9^^,^^14^^,^^43^^,^^59^^,^^79^^,^^100^^,^^106^

There were additional determinants related to the sustainability of the KT process. Fifteen reviews highlighted that embedding evidence within social contexts is essential for achieving system level change within organizations and local communities^25^^,^^30^^,^^31^^,^^35^^,^^40^^,^^42^^,^^59^^,^^65^^,^^66^^,^^67^^,^^69^^,^^85^^,^^97^^,^^110^^,^^111^; although Davies et al.^33^ noted that very few KT strategies targeted the general public. In a study of adaptations in evidence-based interventions, the most cited concern was the need for cultural appropriateness, and acceptability.^35^ Common adaptations included content (100%), context (95.2%), cultural modifications (73.8%) and delivery (61.9%).^35^ This relies on the consolidation of partnerships for health, and outreach to key players in the communities where the interventions would take place. Haynes et al.^112^ note that platforms for cross-sectoral collaborations can increase decision-makers’ capacity to use evidence. This highlights the importance of appraising potential conflicts of interests across stakeholder groups^113^ when KT is the aim.

**Figure 1:**
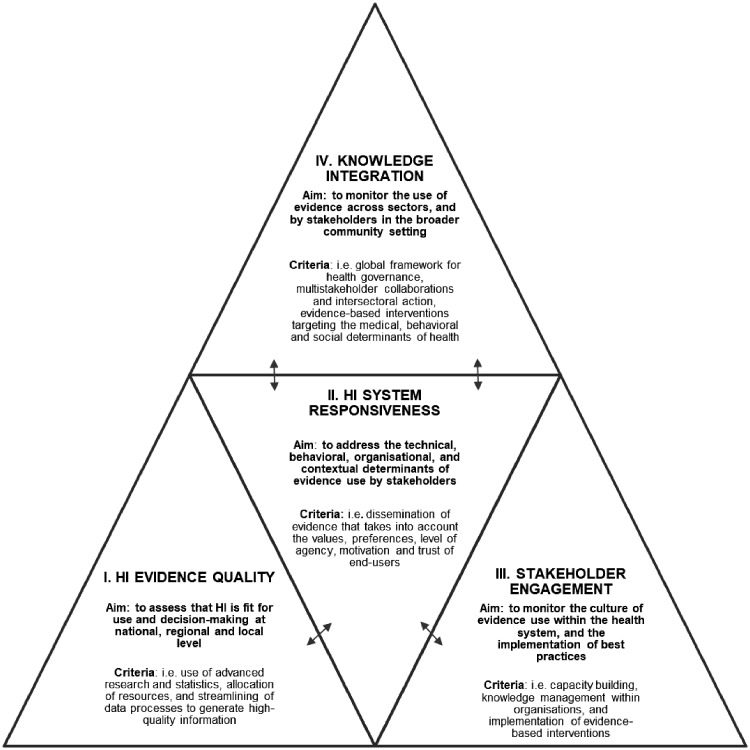
The Health Information (HI)-Impact framework: evaluation domains for monitoring the impact of national health information systems in public health policy and practice. *Notes:* I. Health Information (HI) Evidence Quality relates to data production, accuracy and content relevance; II. HI System Responsiveness reflects the attributes of the wider data infrastructure that enhance the likelihood of information to be used—by improving the end-user experience and addressing expectations on how to access and work with the data; this domain mediates the interaction between the HI systems that generate evidence and the decision-makers in public health policy and practice as represented by the double arrows; III. Stakeholder Engagement relates to the use of HI for training, decision-making and the implementation of interventions in public health policy and practice; IV. Knowledge Integration relates to the use of HI by community partners, in multi-stakeholder coalitions, and for cross-sectoral actions with a broader reach on the determinants of health. This domains builds on the other three domains and furthers the use of HI in all policies for a greater societal impact

Finally, we have synthesized our finding into four domains that are relevant for capturing the mechanisms and determinants that drive KT. The domains of ‘HI Evidence Quality’, ‘HI System Responsiveness’, ‘Stakeholder Engagement’ and ‘Knowledge Integration’ constitute the building blocks of the HI-Impact framework shown in figure 1. These conceptual domains could serve to monitor the impact of national HI systems on reducing the evidence-to-practice gap. More specific evaluation criteria could be developed in each domain to address the quality of the evidence provided by HI systems, the extent of the dissemination efforts (i.e. evaluating ‘Responsiveness’), and how this evidence has influenced decision-making and intervention (i.e. the level of ‘Stakeholder Engagement’ and ‘Knowledge Integration’ across practice fields). We develop further the relevance of these domains for HI systems evaluation in the discussion.

## Discussion

Securing evidence for policy development and intervention is not a strictly linear process. KT relies on feedback and exchange mechanisms between data-providers and data-users working in government, clinics, health agencies and in the broader community. To our knowledge, this study is the first to propose an original framework for monitoring the impact of national HI systems based on lessons from the field of KT. However, we experienced several methodological limitations during the scoping review. As stated by Goldner et al.^37^ and Straus et al.,^22^ the concept of KT is an umbrella term and inconsistencies in the terminology made it more difficult to screen eligible publications. There is a lack of guidance in choosing one KT framework over another, many KT frameworks have not been evaluated, and consensual outcome measures of KT are lacking.^102^^,^^114–116^ Nevertheless, a strength of this scoping review is the breadth of our research, we have examined 79 reviews of KT frameworks encompassing 3916 studies. Furthermore, a new HI systems evaluation framework focusing on impact is highly relevant for Europe given that PRISM was originally intended for strengthening systems in low- and middle-income countries,^15^ and the KT tools from other high-income countries such as the USA or Canada were not intended for the evaluation of national HI systems.

National HI systems and the data they produce are needed to respond to complex health and health system challenges.^24^^,^^117^ However, ensuring that routinely collected data are fit-for-use and decision-making can be a challenge. HI systems collect dispersed data from various sources, including systems which were not initially established for public health surveillance purposes (i.e. administrative and health insurance registries). The quality of routinely collected HI can also vary, and there are noted issues with the ascertainment of causes-of-death statistics for instance across countries. Despite these limitations, routinely collected data are often the best evidence available at population level for decision-makers. Therefore, ensuring that robust and timely evidence is generated and accessed by stakeholders is important, and this is why we have included ‘HI Evidence Quality’ as one of our domains (see figure 1) in line with traditional HI system evaluation frameworks.^1^

The cycle of evidence-informed public health starts with data.^24^ Without it, decision-makers cannot assess the problem or act on it effectively. Yet, the quality of information alone cannot guarantee that it will be used for decision-making.^118^^,^^119^ In public health, there is a lack of global operational guidelines for data sharing.^120^ Therefore, it is also relevant to consider what can be done to facilitate the access to evidence for intervention. ‘Responsiveness’ is a term originally used to describe the quality of the patient–provider dyad and interaction; it encompasses attributes which promote the best patient experience that are linked to quality of care and outcomes (i.e. dignity, respect and autonomy).^121^ Given the importance of dissemination in the KT process, we propose an original application of the concept of ‘Responsiveness’ to HI systems. A responsive HI system would aim to facilitate the access to, and use of HI by lifting the technical, behavioural, organizational and contextual barriers to evidence-use (those listed in table 2).

In our framework, monitoring ‘HI Evidence Quality’, and ‘HI System Responsiveness’ would provide insight on the availability and accessibility of robust and credible evidence. This could entail monitoring whether the evidence provided to stakeholders adheres to the FAIR: findable, accessible, interoperable and reusable data principles.^122^ Anell et al.^123^ also noted that the reporting format has an influence on decision-making, and with ‘league tables, decision-makers tended to suggest more actions compared to funnel plots’ which could be another point of evaluation. Since community mobilization and social marketing approaches have also been recognized as effective KT strategies,^37^ new types of data capture methods (i.e. real-world data from sensors, mobile phones and social media) might also play a role in the evaluation of ‘Responsiveness’. When data are presented in international conferences and high-impact journals, synthesized for decision-makers,^124^ and also presented in plain language summaries, and in social media,^125^ this can increase the impact of HI systems. For example, Euro-Peristat, a European maternal and child health information network uses a wide array of dissemination strategies to reach their stakeholders in 31 countries. Euro-Peristat results are published in comprehensive reports for clinicians and policy-makers, presented in international scientific conferences and peer-reviewed journals, on their project website and in a quarterly newsletter.

Our review of KT determinants also highlighted the importance of organizational readiness for evidence-based practice. Therefore, monitoring HI system use within healthcare facilities, and institutions is the third domain in our framework (figure 1) which we refer to as ‘Stakeholder Engagement’. The increasing use of multidimensional and composite indicators such as the *Healthy Life Years*,^13^ and the *Disability Adjusted Life Years* illustrate the need for HI systems to provide data that resonate with the complex problems that policy-makers face in health.^126^ Yet, the lack of joint understanding can negatively influence the KT process, and the sustainability of evidence-based interventions in the field.^59^^,^^98^ For example in 2018, the French Government pushed for the reduction of the speed limitation from 90 to 80 km/h^−1^ (50 mph) on two-lane highways. Despite data on the number of avoidable deaths (300–400 per year), and an increasing trend in road mortality since 2014, this measure was met with significant resistance from local authorities. Ultimately, national enforcement was amended to voluntary implementation by the regions. (https://www.theguardian.com/world/2018/jan/10/france-cuts-speed-limit-rise-deaths). Politics and external actors with vested interests also influence policies—this can lead to dissonance when objectives across sectors do not align to improve health.^12^^,^^127^ In Europe, the Joint Research Centre, EVIPnet and the European Health Observatory facilitate the continued use of evidence in policy development.

Monitoring the impact of HI systems within the broader social context could provide valuable information on the extent of HI system use. This is because health is largely influenced by determinants outside the health sector as recognized in Dahlgren and Whitehead’s^128^ model of the layers of influence on health, and the more recent focus on ‘Health in all policies’.^86^ Therefore, we have included ‘Knowledge Integration’ in civil society and across sectors as the fourth domain in the HI-Impact framework. This domains relates to the effective incorporation of knowledge into the decisions, practices and policies of organizations and systems.^65^ With the Sustainable Development Goals, countries have been encouraged to build alliances for health in all policies; yet these integrated approaches are currently not reflected in the way national HI systems are being evaluated.^86^ In their review of HI system evaluation frameworks, Eslami et al.^1^ stressed that the role of context has been largely neglected thus far, and requires more attention.

Together, the domains of ‘Stakeholder engagement’ and ‘Knowledge integration’ aim to ensure that HI systems are providing data that are used in coherent interventions on the medical, social and commercial determinants of health. These domains expand the scope of existing HI system evaluation frameworks, by examining the contextual impact of HI systems in areas which might not have been considered before (i.e. across sectors and by the general public). In a recent study, commissioned by the European Parliament, it was noted that ‘faced with emerging threats relating to the spread of disinformation and pseudo-science (…) fostering scientific literacy can provide people with tools to navigate (…) the vast amounts of information exchanged in public debate.’ The inefficient use of healthcare services and poorer health also depends on the level of health knowledge, socio-demographic characteristics and education.^129^^,^^130^ Therefore, increasing scientific and health literacy could constitute another important lever of impact for HI systems.

In conclusion, the field of KT could serve to secure current and future investments in HI systems, by drawing attention to the mechanisms and determinants of evidence-based public health practice and policy-making. In this study, we have answered *how* we might want to evaluate the impact of national HI systems, and *why* the HI-Impact framework domains could be relevant. However, input from public health professionals and policy-makers will be critical to operationalize *what* specifically should be evaluated in each domain. To this aim, we have conducted an online DELPHI exercise with experts in 38 countries, and the development of an evaluation tool based on the HI-Impact framework is currently underway. Further piloting in European public health agencies could contribute to a more balanced assessment of HI systems in each country—one that takes into account the quality of the evidence, as well as the health and societal impact of the systems generating these data.

## Funding

M.D. is funded by a Marie Skłodowska-Curie Action Individual Fellowship, GA No 795051; D.I. is funded by HealthPros, a Marie Skłodowska-Curie Innovative Training Network, and GA No 765141.

*Conflicts of interest*: None declared.

**Key points**

- Creating a culture of accountability for evidence-based decision-making requires close monitoring of health information (HI) use in policy, at the points of care and service delivery, and in the broader community.
- Current frameworks used to evaluate the performance of HI systems focus on data production; we however, encourage giving increased attention to the dissemination, relevance and use of HI in all policies, and by new actors in civil society.
- There should be a deliberate strategy to provide HI to stakeholders within and outside the health system for a greater societal impact.

## Supplementary Material

ckz164_Supplementary_Data
