# Supplementary material for: How can we monitor the impact of national health information systems? Results from a scoping review
Source: Eur J Public Health. 2019 Oct 24;30(4):648–59. doi: 10.1093/eurpub/ckz164 (PMC7445047; doi:10.1093/eurpub/ckz164)
Supplement: ckz164_Supplementary_Data [file ckz164_supplementary_data.zip › ejph-2019-02-srm-0153-File002.docx]

Supplementary References

(2) Rosenkotter N, Achterberg PW, van Bon-Martens MJ, Michelsen K, van Oers HA, Brand H. Key features of an EU health information system: a concept mapping study. Eur J Public Health 2016 Feb;26(1):65-70.

(3) Verschuuren M, van BA, Rosenkotter N, Tijhuis M, Van Oyen H. Towards an overarching European health information system. Eur J Public Health 2017 Oct 1;27(suppl_4):44-8.

(4) Hanney SR, Gonzalez-Block MA. 'Knowledge for better health' revisited - the increasing significance of health research systems: a review by departing Editors-in-Chief. Health Res Policy Syst 2017 Oct 2;15(1):81.

(5) Smith LK, Hindori-Mohangoo AD, Delnord M, Durox M, Szamotulska K, Macfarlane A, et al. Quantifying the burden of stillbirths before 28 weeks of completed gestational age in high-income countries: a population-based study of 19 European countries. Lancet 2018 Nov 3;392(10158):1639-46.

(6) Zeitlin J, Mortensen L, Cuttini M, Lack N, Nijhuis J, Haidinger G, et al. Declines in stillbirth and neonatal mortality rates in Europe between 2004 and 2010: results from the Euro-Peristat project. J Epidemiol Community Health 2016 Jun;70(6):609-15.

(7) Bosetti C, Bertuccio P, Malvezzi M, Levi F, Chatenoud L, Negri E, et al. Cancer mortality in Europe, 2005-2009, and an overview of trends since 1980. Ann Oncol 2013 Oct;24(10):2657-71.

(8) Van Oyen H., Nusselder W, Jagger C, Kolip P, Cambois E, Robine JM. Gender differences in healthy life years within the EU: an exploration of the "health-survival" paradox. Int J Public Health 2013 Feb;58(1):143-55.

(10) WHO Regional Office for Europe. Health 2020. A European policy framework and strategy for the 21st century. Copenhagen, 2013. http://www.euro.who.int/en/publications/abstracts/health-2020.-a-european-policy-framework-and-strategy-for-the-21st-century-2013 accessed December 2018.

(11) Braithwaite J, Marks D, Taylor N. Harnessing implementation science to improve care quality and patient safety: a systematic review of targeted literature. Int J Qual Health Care 2014 Jun;26(3):321-9.

(12) AbouZhar C, Boerma T, Hogan D. Global estimates of country health indicators: useful, unnecessary, inevitable? Global Health Action 2017;10(supp(1): 1290370).

(17) Yost J, Dobbins M, Traynor R, Decorby K, Workentine S, Greco L. Tools to support evidence-informed public health decision making. BMC Public Health 2014 Jul 18;14:728.

(18) WHO Regional Office for Europe. Support tool to assess health information systems and develop and strengthen health information strategies. Copenhagen, 2015. http://www.euro.who.int/en/publications/abstracts/support-tool-to-assess-health-information-systems-and-develop-and-strengthen-health-information-strategies accessed December 2018orld Health Organisation.

(19) Ohkubo S. SSHSV, Timmons BT&SM. Guide to Monitoring and Evaluating Knowledge Management in Global Health Programs. Johns Hopkins Bloomberg School of Public Health; 2013.

(20) Sullivan TM, Strachan M, Timmons BK. Guide to Monitoring and Evaluating Health Information Products and Services. Baltimore,Maryland: Baltimore,Maryland: Center for Communication Programs, Johns Hopkins Bloomberg School of Public Health; Washington, D.C.: Constella Futures; Cambridge, Massachusetts: Management Sciences for Health; 2007. https://www.k4health.org/sites/default/files/guide-to-monitoring-and-evaluating-health-information.pdf accessed October 2018.

(21) The Joanna Briggs Institute. The Joanna Briggs Institute Reviewers' Manual 2015: Methodology for JBI Scoping Reviews. 2019. The Joanna Briggs Institute.

(23) Nilsen P. Making sense of implementation theories, models and frameworks. Implement Sci 2015 Apr 21;10:53.

(24) Vollmar HC, Santos S, de JA, Meyer G, Wilm S. [How does knowledge reach health care practice? : Implementation research and knowledge circulation]. Bundesgesundheitsblatt Gesundheitsforschung Gesundheitsschutz 2017 Oct;60(10):1139-46.

(25) Armstrong R, Waters E, Dobbins M, Anderson L, Moore L, Petticrew M, et al. Knowledge translation strategies to improve the use of evidence in public health decision making in local government: intervention design and implementation plan. Implement Sci 2013 Oct 9;8:121.

(27) Ben CA, Zomahoun HTV, LeBlanc A, Langlois L, Wolfenden L, Yoong SL, et al. Effective strategies for scaling up evidence-based practices in primary care: a systematic review. Implement Sci 2017 Nov 22;12(1):139.

(28) Brown CH, Curran G, Palinkas LA, Aarons GA, Wells KB, Jones L, et al. An Overview of Research and Evaluation Designs for Dissemination and Implementation. Annu Rev Public Health 2017 Mar 20;38:1-22.

(29) Brownson RC, Reis RS, Allen P, Duggan K, Fields R, Stamatakis KA, et al. Understanding administrative evidence-based practices: findings from a survey of local health department leaders. Am J Prev Med 2014 Jan;46(1):49-57.

(30) Burchett H, Umoquit M, Dobrow M. How do we know when research from one setting can be useful in another? A review of external validity, applicability and transferability frameworks. J Health Serv Res Policy 2011 Oct;16(4):238-44.

(32) Darzi A, Abou-Jaoude EA, Agarwal A, Lakis C, Wiercioch W, Santesso N, et al. A methodological survey identified eight proposed frameworks for the adaptation of health related guidelines. J Clin Epidemiol 2017 Jun;86:3-10.

(36) Gardois P, Booth A, Goyder E, Ryan T. Health promotion interventions for increasing stroke awareness in ethnic minorities: a systematic review of the literature. BMC Public Health 2014 Apr 28;14:409.

(37) Goldner EM, Jenkins EK, Fischer B. A narrative review of recent developments in knowledge translation and implications for mental health care providers. Can J Psychiatry 2014 Mar;59(3):160-9.

(38) Hanson RF, Self-Brown S, Rostad WL, Jackson MC. The what, when, and why of implementation frameworks for evidence-based practices in child welfare and child mental health service systems. Child Abuse Negl 2016 Mar;53:51-63.

(39) Jones CA, Roop SC, Pohar SL, Albrecht L, Scott SD. Translating knowledge in rehabilitation: systematic review. Phys Ther 2015 Apr;95(4):663-77.

(40) Leeman J, Calancie L, Hartman MA, Escoffery CT, Herrmann AK, Tague LE, et al. What strategies are used to build practitioners' capacity to implement community-based interventions and are they effective?: a systematic review. Implement Sci 2015 May 29;10:80.

(41) Lourida I, Abbott RA, Rogers M, Lang IA, Stein K, Kent B, et al. Dissemination and implementation research in dementia care: a systematic scoping review and evidence map. BMC Geriatr 2017 Jul 14;17(1):147.

(43) Matus J, Walker A, Mickan S. Research capacity building frameworks for allied health professionals - a systematic review. BMC Health Serv Res 2018 Sep 15;18(1):716.

(44) Meyers DC, Durlak JA, Wandersman A. The quality implementation framework: a synthesis of critical steps in the implementation process. Am J Community Psychol 2012 Dec;50(3-4):462-80.

(46) Moullin JC, Sabater-Hernandez D, Fernandez-Llimos F, Benrimoj SI. A systematic review of implementation frameworks of innovations in healthcare and resulting generic implementation framework. Health Res Policy Syst 2015 Mar 14;13:16.

(47) Payne PR, Embi PJ, Sen CK. Translational informatics: enabling high-throughput research paradigms. Physiol Genomics 2009 Nov 6;39(3):131-40.

(48) Rajan A, Sullivan R, Bakker S, van Harten WH. Critical appraisal of translational research models for suitability in performance assessment of cancer centers. Oncologist 2012;17(12):e48-e57.

(50) Slade SC, Philip K, Morris ME. Frameworks for embedding a research culture in allied health practice: a rapid review. Health Res Policy Syst 2018 Mar 21;16(1):29.

(51) Stirman SW, Gutner CA, Langdon K, Graham JR. Bridging the Gap Between Research and Practice in Mental Health Service Settings: An Overview of Developments in Implementation Theory and Research. Behav Ther 2016 Nov;47(6):920-36.

(53) van der Veer SN, Jager KJ, Nache AM, Richardson D, Hegarty J, Couchoud C, et al. Translating knowledge on best practice into improving quality of RRT care: a systematic review of implementation strategies. Kidney Int 2011 Nov;80(10):1021-34.

(55) Welch VA, Petticrew M, O'Neill J, Waters E, Armstrong R, Bhutta ZA, et al. Health equity: evidence synthesis and knowledge translation methods. Syst Rev 2013 Jun 22;2:43.

(58) Hack TF, Carlson L, Butler L, Degner LF, Jakulj F, Pickles T, et al. Facilitating the implementation of empirically valid interventions in psychosocial oncology and supportive care. Support Care Cancer 2011 Aug;19(8):1097-105.

(59) Kneale D, Rojas-Garcia A, Raine R, Thomas J. The use of evidence in English local public health decision-making: a systematic scoping review. Implement Sci 2017 Apr 20;12(1):53.

(60) Leeman J, Birken SA, Powell BJ, Rohweder C, Shea CM. Beyond "implementation strategies": classifying the full range of strategies used in implementation science and practice. Implement Sci 2017 Nov 3;12(1):125.

(61) Stander J, Grimmer K, Brink Y. Training programmes to improve evidence uptake and utilisation by physiotherapists: a systematic scoping review. BMC Med Educ 2018 Jan 15;18(1):14.

(62) Ward V, House A, Hamer S. Developing a framework for transferring knowledge into action: a thematic analysis of the literature. J Health Serv Res Policy 2009 Jul;14(3):156-64.

(63) Wilson CL, Johnson D, Oakley E. Knowledge translation studies in paediatric emergency medicine: A systematic review of the literature. J Paediatr Child Health 2016 Feb;52(2):112-25.

(64) Wilson PM, Petticrew M, Calnan MW, Nazareth I. Disseminating research findings: what should researchers do? A systematic scoping review of conceptual frameworks. Implement Sci 2010 Nov 22;5:91.

(65) Best A, Terpstra JL, Moor G, Riley B, Norman CD, Glasgow RE. Building knowledge integration systems for evidence-informed decisions. J Health Organ Manag 2009;23(6):627-41.

(66) Garside R, Pearson M, Moxham T. What influences the uptake of information to prevent skin cancer? A systematic review and synthesis of qualitative research. Health Educ Res 2010 Feb;25(1):162-82.

(68) Novins DK, Green AE, Legha RK, Aarons GA. Dissemination and implementation of evidence-based practices for child and adolescent mental health: a systematic review. J Am Acad Child Adolesc Psychiatry 2013 Oct;52(10):1009-25.

(71) Messina J, Campbell S, Morris R, Eyles E, Sanders C. A narrative systematic review of factors affecting diabetes prevention in primary care settings. PLoS One 2017;12(5):e0177699.

(73) Phillipson L, Goodenough B, Reis S, Fleming R. Applying Knowledge Translation Concepts and Strategies in Dementia Care Education for Health Professionals: Recommendations From a Narrative Literature Review. J Contin Educ Health Prof 2016;36(1):74-81.

(74) Bish A, Yardley L, Nicoll A, Michie S. Factors associated with uptake of vaccination against pandemic influenza: a systematic review. Vaccine 2011 Sep 2;29(38):6472-84.

(75) Turner S, D'Lima D, Hudson E, Morris S, Sheringham J, Swart N, et al. Evidence use in decision-making on introducing innovations: a systematic scoping review with stakeholder feedback. Implement Sci 2017 Dec 4;12(1):145.

(76) Dogherty EJ, Harrison M, Graham I, Keeping-Burke L. Examining the use of facilitation within guideline dissemination and implementation studies in nursing. Int J Evid Based Healthc 2014 Jun;12(2):105-27.

(77) Bussieres AE, Al ZF, Stuber K, French SD, Boruff J, Corrigan J, et al. Evidence-based practice, research utilization, and knowledge translation in chiropractic: a scoping review. BMC Complement Altern Med 2016 Jul 13;16:216.

(78) Wandersman A, Chien VH, Katz J. Toward an evidence-based system for innovation support for implementing innovations with quality: tools, training, technical assistance, and quality assurance/quality improvement. Am J Community Psychol 2012 Dec;50(3-4):445-59.

(80) Shahmoradi L, Safadari R, Jimma W. Knowledge Management Implementation and the Tools Utilized in Healthcare for Evidence-Based Decision Making: A Systematic Review. Ethiop J Health Sci 2017 Sep;27(5):541-58.

(82) Welch V, Tugwell P, Morris EB. The equity-effectiveness loop as a tool for evaluating population health interventions. Rev Salud Publica (Bogota ) 2008 Dec;10 Suppl:83-96.

(83) Shawky S. Measuring Geographic and Wealth Inequalities in Health Distribution as Tools for Identifying Priority Health Inequalities and the Underprivileged Populations. Glob Adv Health Med 2018;7:2164956118791955.

(84) Perez-Lu JE, Bayer AM, Iguiniz-Romero R. Information = equity? How increased access to information can enhance equity and improve health outcomes for pregnant women in Peru. J Public Health (Oxf) 2018 Dec 1;40(suppl_2):ii64-ii73.

(88) Weiss D, Lillefjell M, Magnus E. Facilitators for the development and implementation of health promoting policy and programs - a scoping review at the local community level. BMC Public Health 2016 Feb 11;16:140.

(89) Bennett C. Up the hierarchy. Journal of Extension 1975;7-12.

(91) Glasgow RE, Klesges LM, Dzewaltowski DA, Estabrooks PA, Vogt TM. Evaluating the impact of health promotion programs: using the RE-AIM framework to form summary measures for decision making involving complex issues. Health Educ Res 2006 Oct;21(5):688-94.

(93) Budrionis A, Bellika JG. The Learning Healthcare System: Where are we now? A systematic review. J Biomed Inform 2016 Dec;64:87-92.

(94) Barac R, Stein S, Bruce B, Barwick M. Scoping review of toolkits as a knowledge translation strategy in health. BMC Med Inform Decis Mak 2014 Dec 24;14:121.

(95) Gagnon MP, Attieh R, Ghandour eK, Legare F, Ouimet M, Estabrooks CA, et al. A systematic review of instruments to assess organizational readiness for knowledge translation in health care. PLoS One 2014;9(12):e114338.

(99) Santos M, Eriksson H. Making quality registers supporting improvements: a systematic review of the data visualization in 5 quality registries. Qual Manag Health Care 2014 Apr;23(2):119-28.

(100) Haynes A, Rowbotham SJ, Redman S, Brennan S, Williamson A, Moore G. What can we learn from interventions that aim to increase policy-makers' capacity to use research? A realist scoping review. Health Res Policy Syst 2018 Apr 10;16(1):31.

(102) Allen JD, Towne SD, Jr., Maxwell AE, DiMartino L, Leyva B, Bowen DJ, et al. Meausures of organizational characteristics associated with adoption and/or implementation of innovations: A systematic review. BMC Health Serv Res 2017 Aug 23;17(1):591.

(103) Wye L, Brangan E, Cameron A, Gabbay J, Klein JH, Pope C. Evidence based policy making and the 'art' of commissioning - how English healthcare commissioners access and use information and academic research in 'real life' decision-making: an empirical qualitative study. BMC Health Serv Res 2015 Sep 29;15:430.

(104) Oliver KA, de VF, Money A, Everett M. Identifying public health policymakers' sources of information: comparing survey and network analyses. Eur J Public Health 2017 May 1;27(suppl_2):118-23.

(105) Oliver KA, de Vocht F. Defining 'evidence' in public health: a survey of policymakers' uses and preferences. Eur J Public Health 2017 May 1;27(suppl_2):112-7.

(107) Mutale W, Chintu N, Amoroso C, Awoonor-Williams K, Phillips J, Baynes C, et al. Improving health information systems for decision making across five sub-Saharan African countries: Implementation strategies from the African Health Initiative. BMC Health Serv Res 2013;13 Suppl 2:S9.

(109) De Angelis G, Davies B, King J, McEwan J, Cavallo S, Loew L, et al. Information and Communication Technologies for the Dissemination of Clinical Practice Guidelines to Health Professionals: A Systematic Review. JMIR Med Educ 2016 Nov 30;2(2):e16.

(110) Heijmans N, van Lieshout J, Wensing M. Information exchange networks of health care providers and evidence-based cardiovascular risk management: an observational study. Implement Sci 2017 Jan 13;12(1):7.

(111) Jylha V, Mikkonen S, Saranto K, Bates DW. The Impact of Information Culture on Patient Safety Outcomes. Development of a Structural Equation Model. Methods Inf Med 2017 Mar 8;56(Open):e30-e38.

(112) Haynes A, Rowbotham SJ, Redman S, Brennan S, Williamson A, Moore G. What can we learn from interventions that aim to increase policy-makers' capacity to use research? A realist scoping review. Health Res Policy Syst 2018 Apr 10;16(1):31.

(113) Whitney W, Dutcher GA, Keselman A. Evaluation of health information outreach: theory, practice, and future direction. J Med Libr Assoc 2013 Apr;101(2):138-46.

(114) Birken SA, Powell BJ, Shea CM, Haines ER, Alexis KM, Leeman J, et al. Criteria for selecting implementation science theories and frameworks: results from an international survey. Implement Sci 2017 Oct 30;12(1):124.

(115) Lynch EA, Mudge A, Knowles S, Kitson AL, Hunter SC, Harvey G. "There is nothing so practical as a good theory": a pragmatic guide for selecting theoretical approaches for implementation projects. BMC Health Serv Res 2018 Nov 14;18(1):857.

(116) Potvin L. Wishful thinking will not do it! Practitioners and decision-makers need tools to implement evidence-informed public health. Int J Public Health 2013 Aug;58(4):491-2.

(117) Bogaert P, Van Oyen H. An integrated and sustainable EU health information system: national public health institutes' needs and possible benefits. Arch Public Health 2017;75:3.

(118) NICE uptake and impact report: A biannual report on the uptake of NICE products and NICE's impact on health and social care. UK, 2017. https://www.nice.org.uk/Media/Default/About/what-we-do/Into-practice/measuring-uptake/nice-uptake-and-impact-report-mar-17.pdf accessed 10 October 2018.

(119) Rangachari P, Rissing P, Rethemeyer K. Awareness of evidence-based practices alone does not translate to implementation: insights from implementation research. Qual Manag Health Care 2013 Apr;22(2):117-25.

(121) Mirzoev T, Kane S. What is health systems responsiveness? Review of existing knowledge and proposed conceptual framework. BMJ Glob Health 2017;2(4):e000486.

(122) Wilkinson MD, Dumontier M, Aalbersberg IJ, Appleton G, Axton M, Baak A, et al. The FAIR Guiding Principles for scientific data management and stewardship. Sci Data 2016 Mar 15;3:160018.

(123) Anell A, Hagberg O, Liedberg F, Ryden S. A randomized comparison between league tables and funnel plots to inform health care decision-making. Int J Qual Health Care 2016 Dec 1;28(6):816-23.

(124) Courtney DB, Bennett K, Szatmari P. The Forest and the Trees: Evidence-Based Medicine in the Age of Information. J Am Acad Child Adolesc Psychiatry 2019 Jan;58(1):8-15.

(125) Freudenberg N, Tsui E. Evidence, power, and policy change in community-based participatory research. Am J Public Health 2014 Jan;104(1):11-4.

(126) Bhattacharya D, Bhatt J. Seven Foundational Principles of Population Health Policy. Popul Health Manag 2017 Oct;20(5):383-8.

(128) Dalhren G, Whitehead M. European strategies for tackling social inequities in health: Levelling up Part 2. 2018.

(130) Tille F, Rottger J, Gibis B, Busse R, Kuhlmey A, Schnitzer S. Patients' perceptions of health system responsiveness in ambulatory care in Germany. Patient Educ Couns 2019 Jan;102(1):162-71.

(131) Leeman J, Calancie L, Kegler MC, Escoffery CT, Herrmann AK, Thatcher E, et al. Developing Theory to Guide Building Practitioners' Capacity to Implement Evidence-Based Interventions. Health Educ Behav 2017 Feb;44(1):59-69.

(132) Wallace J, Byrne C, Clarke M. Improving the uptake of systematic reviews: a systematic review of intervention effectiveness and relevance. BMJ Open 2014 Oct 16;4(10):e005834.

(133) van Bon-Martens MJH, van de Goor IAM, van Oers HAM. Concept mapping as a method to enhance evidence-based public health. Eval Program Plann 2017 Feb;60:213-28.
