# Supplementary material for: How can we monitor the impact of national health information systems? Results from a scoping review
Source: Eur J Public Health. 2019 Oct 24;30(4):648–59. doi: 10.1093/eurpub/ckz164 (PMC7445047; doi:10.1093/eurpub/ckz164)
Supplement: ckz164_Supplementary_Data [file ckz164_supplementary_data.zip › ejph-2019-02-srm-0153-File003.docx]

Annex 1 Search strategy for reviews on knowledge translation published in 2008-2018

We searched Pubmed, Medline, and the Cochrane Database of Systematic Reviews between August 2018 - December 2018, using the following key words and MeSh terms combined, and truncated*:

*knowledge*, translation, health data*, dissemination, implementation, adaptation, evidence informed*, evidence based*, public health, health policy, health care, impact, health system*, performance, health information*, tool, framework, monitoring, evaluation*

Eligibility criteria:

**Inclusion criteria:**

- Reviews of KT frameworks/strategies or tools that guide evidence into the hands of health policy makers, healthcare professionals, community managers, patients and their advocates for decision-making.
- Dissemination is defined as a targeted distribution of information.
- Implementation is defined as the use of strategies to integrate evidence into practice.
- Process models (i.e. stages), and determinant frameworks (i.e. facilitators, barriers and challenges) for knowledge translation^1^
- Abstract published in English in the last 10 years, between 2008 and 2018

1. Nilsen P. Making sense of implementation theories, models and frameworks. Implement.Sci. 2015;10:53.

**Exclusion criteria:**

- Evaluations studies and interventions
- Health promotion interventions (secondary outputs of knowledge translation strategies)
- Study not conducted in a high-income setting published in English within the last 10 years.
- Study protocols, commentaries, editorials and opinion pieces
- Full-text article could not be retrieved.
- Reviews or studies which only pertained to individual level change and data uptake.

Results

- (evidence informed) OR (evidence based) AND (public health OR policy OR medicine) AND (framework OR model or theory OR strategies),
   **filters 1**: “Review”, “Systematic Reviews”, “Abstract”, “Humans”, “English”:14127 hits; **filters 2**: “published in last 10 years” 9695; **filter 3**: “ Subjects: systematic review: 2969 hits
  MeSH Terms: policy; public health; medicine
  Additional terms: health; theory; framework; informed; public health; public; model; strategies; policy; medicine; based; evidence
- (translational research OR knowledge translation OR evidence to practice) AND (framework OR model OR theory) AND (public health OR health promotion OR medicine OR policy), **filters**: “Review”, “Systematic Reviews”,” Abstract”, “published in the last years”, “Humans”, ”English”, “Subjects: systematic reviews”: 902 hits

MeSH Terms: translational medical research; medicine; public health; health promotion; policy

Journal: jid101570800; practice (birm)

- Knowledge management frameworks review , **filter**: “Systematic review”,”Published in the last 10 years”: 112 hits
  MeSH Terms: review literature as topic; knowledge management ; Publication Type: review”
- Knowledge translation frameworks, **filter** “Review”, “Systematic review”, “Abstracts”,”published in the last 10 years”: 65 hits
  MeSH Terms: translational medical research
- decision making public health policy framework, **filters**: “Review”, “Systematic review”, “Abstracts”, “Humans”, “English”, “Published in the last 10 years”: 148 hits

MeSH Terms: public health; policy; decision making

- "uptake of evidence", **filters**: “Review”, “Systematic Reviews”, “Abstract”, “published in the last 10 years”, “Humans”, “English”, “Subject: Systematic Reviews”: 599 hits
- (knowledge translation) AND (public health data), **filters**: “Review”, “Systematic Reviews”,” Abstract”, “published in the last 10 years”, “Humans”, ”English”, “Subjects: systematic reviews”: 231 hits

MeSH Terms: translational medical research; public health

Journal: jid101699766; jid8904655; data (basel); brown univ dig addict theory appl

Additional terms: public health; translational; translation; research; health; medical; knowledge; public; translational medical research; knowledge translation; data

- (knowledge translation) AND (uptake) AND (theory), filter: “Reviews”,” “Systematic Reviews”, ”Abstract”, “published in the last 10 years”, “Humans”, ”English”: 4 hits

MeSH Terms: translational medical research

Additional terms: knowledge; uptake; theory; knowledge translation; translational; medical; translation; research; translational medical research

- (health information) AND (use) AND (practice) AND (framework), **filter**: “Review, Systematic Reviews, published in the last 10 years, Humans, English”: 74 hits
  Subheading: statistics and numerical data

MeSH Terms: health

Journal: information (basel); practice (birm)

Additional terms: practice; framework; health; information; data; statistics; statistics and numerical data; numerical; use

- evaluating impact of health information systems, **filters**: “Review, Systematic Reviews, published in the last 10 years, Humans, English”: 38 hits

MeSH Terms: health information systems

Journal: impact (am coll physicians)

- (health information system) AND ( country) AND (evaluation), **filters**:” Review, Systematic Reviews, published in the last 10 years, Humans, English.”: 46 hits
  MeSH Terms: health information systems
- (health information system) AND ( country) AND (evaluation) AND (tool), **filters**:” Review, Systematic Reviews, published in the last 10 years, Humans, English: 3 hits
  MeSH Terms: health information systems
- (routine health information system ) AND (evaluation) AND (tool), **filters**:” Review, Systematic Reviews, published in the last 10 years, Humans, English : 9 hits
  MeSH Terms: health information systems

Journal: jid0415527; jid101597976; evaluation; evaluation (lond)
